# Peripheral and Cerebral Resistance Arteries in the Spontaneously Hypertensive Heart Failure Rat: Effects of Stilbenoid Polyphenols

**DOI:** 10.3390/molecules22030380

**Published:** 2017-02-28

**Authors:** Danielle I. Lee, Crystal Acosta, Christopher M. Anderson, Hope D. Anderson

**Affiliations:** 1 College of Pharmacy, Rady Faculty of Health Sciences, University of Manitoba, 750 McDermot Avenue, Winnipeg, MB R3E 0T5, Canada; dlee@sbrc.ca; 2 Canadian Centre for Agri-Food Research in Health and Medicine, St. Boniface Hospital Research Centre, 351 Taché Avenue, Winnipeg, MB R2H 2A6, Canada; cacosta@sbrc.ca; 3 Department of Pharmacology and Therapeutics, Rady Faculty of Health Sciences, University of Manitoba, 753 McDermot Avenue, Winnipeg, MB R3E 0T6, Canada; chris.anderson@umanitoba.ca; 4 Neuroscience Research Program, Kleysen Institute for Advanced Medicine, Health Sciences Centre, Winnipeg, MB R3E 0Z3, Canada

**Keywords:** resistance arteries, compliance, remodeling, resveratrol, polyphenol, stilbenoid

## Abstract

Hypertension is associated with aberrant structure and mechanical properties of resistance arteries. We determined the effects of resveratrol, a non-flavonoid polyphenol found in foods such as red grapes, and structurally-similar analogues (pterostilbene and gnetol) on systolic blood pressure (SBP) and resistance arteries from the spontaneously hypertensive heart failure (SHHF) rat. SBP was elevated in 17-week-old SHHF vs. Sprague-Dawley rats (normotensive control; 194 ± 3 vs. 142 ± 6 mmHg, *p* < 0.01) and was unaffected by resveratrol, pterostilbene, or gnetol (2.5 mg/kg/d). Geometry and mechanical properties of pressurized mesenteric resistance arteries and middle cerebral arteries were calculated from media and lumen dimensions measured at incremental intraluminal pressures. SHHF arteries exhibited remodeling which consisted of augmented media-to-lumen ratios, and this was attenuated by stilbenoid treatment. Compliance was significantly reduced in SHHF middle cerebral arteries but not mesenteric arteries vis-à-vis increased wall component stiffness; stilbenoid treatment failed to normalize compliance and wall component stiffness. Our data suggest that neither AMPK nor ERK mediate stilbenoid effects. In conclusion, we observed arterial bed-specific abnormalities, where mesenteric resistance arteries exhibited remodeling and cerebral arteries exhibited remodeling and stiffening. Resveratrol, pterostilbene, and gnetol exhibited similar abilities to attenuate vascular alterations.

## 1. Introduction

Blood pressure is proportional to cardiac output and total peripheral resistance. Since resistance arteries by definition generate resistance to blood flow, and abnormalities of resistance arteries play a role in the pathogenesis and maintenance of hypertension in humans and experimental animals [1,2]. As resistance to blood flow is inversely proportional to the fourth power of the arterial lumen radius, it is important to understand how lumen diameters of these arteries are modified when studying the pathogenesis and prevention or reversal of hypertension. In the spontaneously hypertensive rat (SHR), mesenteric resistance arteries undergo a combination of hypertrophic and eutrophic remodeling, which are typically characterized by increased media-to-lumen ratios [3,4,5]. Moreover, changes in the mechanical properties or stiffness of an artery can also influence the degree of resistance by affecting pressure-lumen diameter relationships [6].

Epidemiological evidence suggests that healthy dietary approaches can reduce cardiovascular morbidity. The 8-week Dietary Approaches to Stop Hypertension (DASH) trial demonstrated that the combination of increased fruit, vegetable, and fish consumption with reductions in saturated fat intake reduced blood pressure in hypertensive patients [7]. Likewise, the Lyon Diet Heart Study showed that dietary intervention comprised of increased consumption of fruit, vegetables, fish, as well as the omega-3 fatty acid, alpha-linolenic acid, led to a significant reduction in cardiovascular morbidity and prolonged survival following myocardial infarction [8,9]. As reviewed by Dohadwala & Vita [10], there is also extensive evidence derived from epidemiological studies suggesting that the polyphenol compounds commonly found in grapes, such as flavonoids, phenolic acids, and resveratrol, reduce cardiovascular mortality [11,12,13,14,15,16]. Cognizant of the contribution of resistance arteries to the etiology of hypertension, McCall et al. conducted a clinical trial to quantify the vascular effects of a fruit and vegetable-based intervention in hypertensive patients where indeed, increased fruit and vegetable consumption dose-dependently improved micro-vascular function [17]. This report did not specifically document which fruit and/or vegetables were consumed. However, it is known that grape-derived foods such as red wine and grape juice can alleviate hypertension in rats and humans [18,19,20,21,22,23]. Given the importance of resistance arteries in determining blood pressure, we were intrigued by the notion that perhaps a bioactive component within grapes might be beneficial to microvascular health. This gave rise to our reported postulate that naturally-occurring stilbenoids, and in particular resveratrol, might be conferring the beneficial effects on microvascular function observed with increased fruit and vegetable consumption [17].

Stilbenoids refer to a class of naturally-occurring bioactive polyphenolic compounds. Resveratrol (*trans*-3,5,4′-trihydroxystilbene) is one such stilbenoid that has been linked to protective effects in cardiovascular and neurodegenerative disease, as well as enhanced longevity [24,25,26,27]. In fact, resveratrol is purportedly one of the components within grapes and red wine that might confer the benefits of moderate red wine consumption on reducing cardiovascular disease risk [28,29]. Indeed, resveratrol is known to reduce blood pressure in a number of animal models with hypertension including partially nephrectomised rats [30], fructose fed rats [31], high-fat diet rats [32], and SHR [33]. We reported that chronic treatment with resveratrol prevented the development of abnormal resistance artery geometry in SHR. However, resveratrol failed to correct abnormalities related to resistance artery wall stiffness [33].

Despite the significant interest, resveratrol has several limitations: it exhibits poor solubility, is readily metabolized and undergoes rapid glucuronidation resulting in low oral bioavailability of ~20%, and half-life of ~14 min [34,35,36]. Therefore, we queried whether alternate stilbenoids might improve upon the therapeutic potential of resveratrol. Pterostilbene (*trans*-3,5-dimethoxy-4′-hydroxystilbene) and gnetol (*trans*-2,6,3′,5′-tetrahydroxystilbene) are two structural derivatives of resveratrol that share the characteristic stilbenoid structure, but minor structural differences that markedly alter their pharmacokinetic and pharmacodynamic profiles. Pterostilbene, a dimethoxylated analogue of resveratrol, is found predominantly in blueberries and in certain species of grapes [37]. A methoxy group occupying position 3 of the benzene ring limits glucuronidation resulting in a reported oral bioavailability of 80% with a half-life of 105 min [36]; thus, we postulated that pterostilbene in particular might exert larger vasculoprotective effects. There is little information on the cardiovascular effects of pterostilbene, other than its ability to inhibit vascular smooth muscle cell migration [38] and enhance blood pressure lowering in patients with high cholesterol [39] (perhaps vis-à-vis anti-inflammatory [40] and anti-oxidant activities [41]). Gnetol has a tetrahydroxy stilbenoid structure and is a naturally-occurring compound in plants of the genus *Gnetum* [42]. In Southeast Asia, seeds and fruit of melinjo (*G. gnemon*) are consumed as functional foods [43]. Melinjo seed extracts and gnetol *per se* are found in traditional Asian medicines and have also been used in NHPs [44,45]. Compared to resveratrol, gnetol has improved solubility but a lower bioavailability (6.59%) [42]. Despite its historical use as a traditional medicine, gnetol effects on cardiovascular health are unknown. We do know that pterostilbene and *G. gnemon* exhibit anti-inflammatory, anti-cancer and anti-oxidant properties, all of which are similar to resveratrol [36,45,46].

Here, we examined the effects of resveratrol, pterostilbene and gnetol on blood pressure and microvasculature in the spontaneously hypertensive heart failure (SHHF) rat. In contrast to SHR which model hypertension, the SHHF rat models human heart disease in that it superimposes risk of heart failure upon hypertension [47,48]. We also probed AMP-activated protein kinase (AMPK) and ERK as potential targets of stilbenoids in SHHF arteries. AMPK is a serine/threonine kinase that acts as a cellular energy sensor, and in SHR aorta, basal AMPK activation status was reduced to approximately 50% of WKY levels. Moreover, an AMPK activator (AICAR or 5-aminoimidazole-4-carboxyamide-1-[β]-d-ribofuranoside) reduced blood pressure and elicited vasorelaxation in mesenteric arteries [49]. Finally, resveratrol effects on the hypertrophied heart are reportedly attributable to effects on AMPK signaling [50]. ERK is another well-documented target of resveratrol. Inhibition of ERK is a major action of resveratrol in aortic [51] and coronary artery smooth muscle cells [52]. ERK plays an important role in vascular remodeling; El Mabrouk et al. reported exaggerated ERK signaling in mesenteric resistance arteries from adult SHR, and growth responses in vascular smooth muscle cells isolated from SHR were blocked by inhibition of ERK [53]. We then reported that the ability of resveratrol to attenuate increased compliance of mesenteric arteries in SHR was associated with complete normalization of ERK to WKY levels [33]. Thus, we speculated that ERK was an important candidate mediator of stilbenoid effects.

## 2. Results

### 2.1. Body Weight and Blood Pressure

At the end of the study (i.e., 17 weeks of age), SD rats exhibited greater body weights compared to SHHF rats (564.3 ± 14.5 vs. 374.7 ± 9.8 g; *p* < 0.01; Table 1). Elevated SBPs (194 ± 3 mm Hg) were observed in SHHF rats compared to the normotensive control rats (SD—142 ± 6 mm Hg; *p* < 0.01; Table 1). No statistically significant effects of stilbenoids on body weight nor SBP were observed.

### 2.2. Vascular Geometry

**Figure 1:**
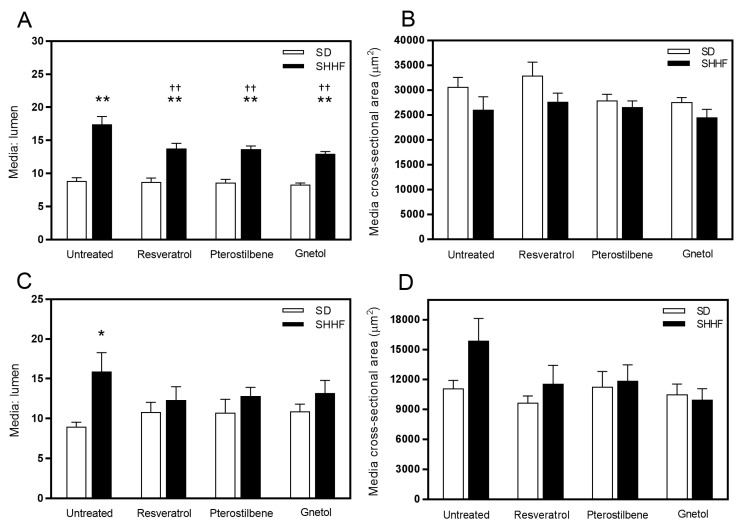
Stilbenoid effects on vascular geometry. Media-to-lumen ratio in (**a**) mesenteric resistance arteries and (**c**) middle cerebral arteries were increased in untreated SHHF rats, and this was attenuated by 8-week treatment with resveratrol, pterostilbene, and gnetol (2.5 mg/kg/day). In contrast, no significant differences in media CSA (**b**,**d**) were detected. *n* = 4–8. * *p* < 0.05 and ** *p* < 0.01 vs. untreated SD. ^††^ *p* < 0.01 vs. untreated SHHF.

Increased media-to-lumen ratios were observed in both mesenteric resistance (Figure 1A) and middle cerebral (Figure 1C) arteries from untreated SHHF rats, whereas despite opposing trends, there were no significant changes in media CSA (Figure 1B,D). Resveratrol, pterostilbene, and gnetol equivalently attenuated increases in media-to-lumen ratios in both mesenteric and cerebral vessels. Mesenteric resistance arteries from untreated SHHF rats exhibited remodeling and growth indices of 97.4% and 3.9%, respectively, whereas middle cerebral arteries exhibited remodeling and growth indices of 58.0% and 43.6%, respectively (Table 2).

### 2.3. Vascular Compliance

**Figure 2:**
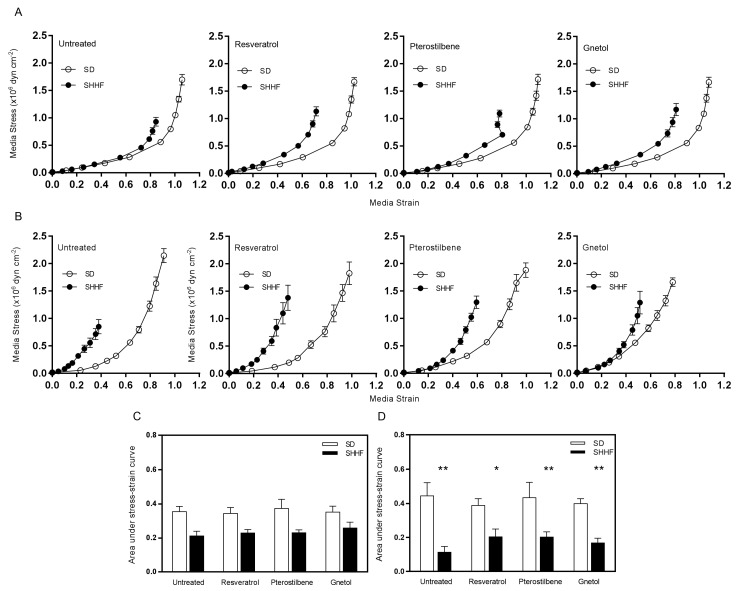
Effect of stilbenoids on vascular compliance. The stress-strain curve of the untreated SHHF mesenteric arteries appears to be shifted to the left (**A**); but when quantified as AUC (**C**); is not statistically significant (*p* = 0.07). Middle cerebral arteries also exhibit a leftward shift of the stress strain curve (**B**); and AUC is significantly reduced (**D**). 8-week treatment with resveratrol, pterostilbene, and gnetol (2.5 mg/kg/day) failed to restore the stress-strain relationship towards normal. (*n* = 4–8). * *p* < 0.05 and ** *p* < 0.01 vs. untreated SD.

Vascular compliance is measured by plotting the relationship between media stress and media strain. Leftward shifts of the stress-strain curve were observed in mesenteric resistance (Figure 2A) and middle cerebral (Figure 2B) arteries from untreated SHHF rats, and this was quantified as decreases in the areas under the curve (AUC) that approached (mesenteric arteries; *p* = 0.07) or achieved statistical significance (cerebral arteries; *p* < 0.01; Figure 2C,D, respectively). Stilbenoid treatment failed to improve compliance in SHHF cerebral arteries.

### 2.4. Arterial Wall Component Stiffness

**Figure 3:**
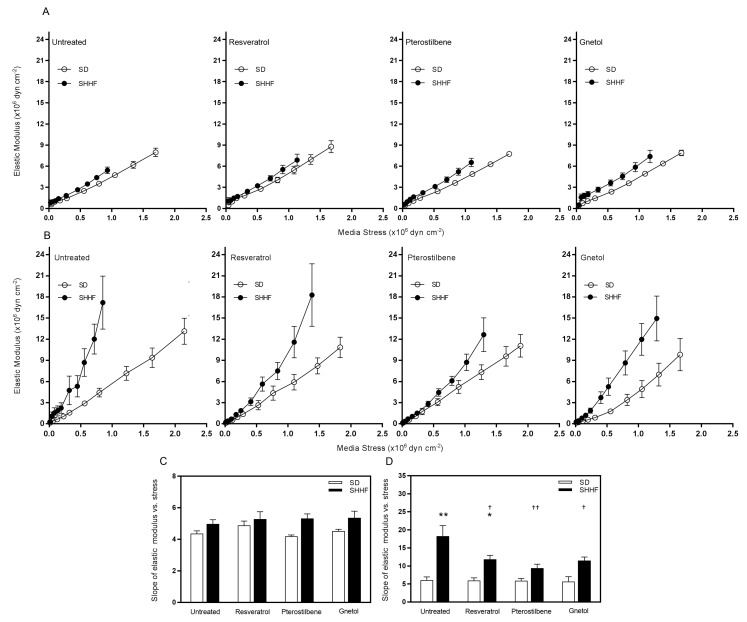
Effect of stilbenoids on arterial wall component stiffness. (**A**) In mesenteric arteries, the elastic modulus-stress curve of untreated SHHF is unchanged relative to untreated SD, and this is reflected by (**C**) similar slopes of the elastic modulus-stress curve between SD and SHHF arteries, in the presence and absence of stilbenoid treatment; (**B**) In middle cerebral arteries, the elastic modulus-stress curve for untreated SHHF is shifted leftward, and this is reflected by (**D**) the statistically significant increase in slope. This was attenuated by 8-week treatment with resveratrol, pterostilbene, and gnetol (2.5 mg/kg/day). *n* = 4–8. * *p* < 0.05 and ** *p* < 0.01 vs. untreated SD. ^†^ *p* < 0.05 and ^††^ *p* < 0.01 vs. untreated SHHF.

Vascular geometry is mathematically eliminated as a determinant of vascular wall stiffness when elastic modulus (EM) is plotted against media stress; therefore, the slope of the EM vs. stress curve provides information pertaining to the stiffness of wall components (such as elastin, collagen, and smooth muscle cells). Although wall component stiffness was similar between SD and SHHF mesenteric arteries (Table 1; Figure 3A,C), it was significantly increased in SHHF middle cerebral arteries (Table 1; Figure 3B,D; *p* < 0.01). Resveratrol, pterostilbene, and gnetol reduced wall component stiffness toward normal (Table 1; Figure 3B,D).

### 2.5. Signaling Effectors

**Figure 4:**
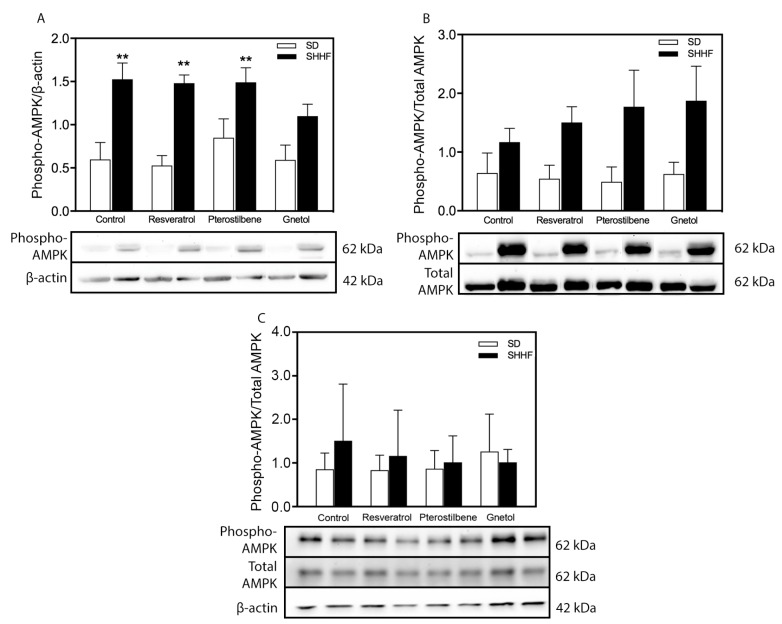
Arterial AMPKα activation status: effect of stilbenoid treatment. Activation of AMPKα vis-à-vis phosphorylation at Thr172 in (**A**) mesenteric arteries from SHHF rats compared to SD arteries is likely due in part to (**B**) a trend where total AMPK levels are increased in untreated and resveratrol- or pterostilbene-treated SHHF rats (since statistical significant changes are obscured by normalization with total AMPK). Only in the presence of gnetol (**A**) was there a lack of statistically-significant AMPKα phosphorylation in SHHF mesenteric arteries. (**C**) No significant differences in AMPK were detected in cerebral arteries. *n* = 3–4. ** *p* < 0.01 vs. SD-C.

**Figure 5:**
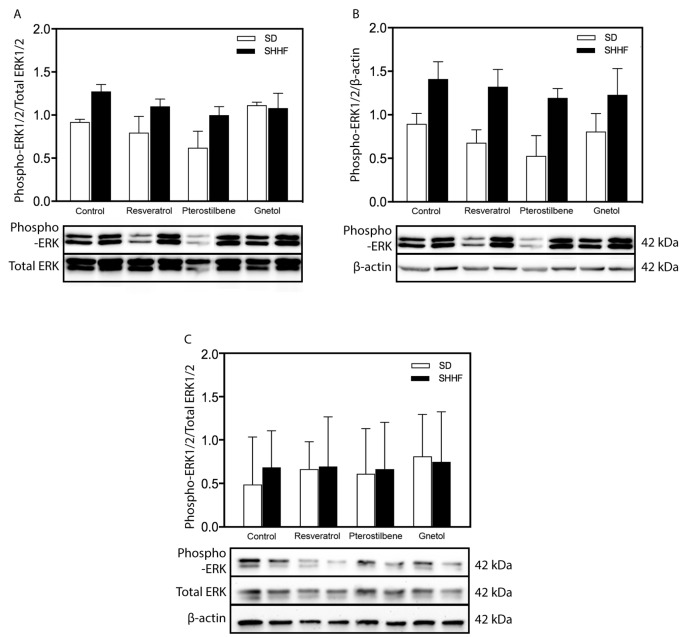
Arterial ERK activation status: effect of stilbenoid treatment. ERK activity was similar in mesenteric arteries whether normalized by (**A**) total ERK; or (**B**) β-actin; and (**C**) cerebral arteries from SHHF rats compared to SD arteries, whether in the presence or absence of stilbenoid treatment. *n* = 3–4.

As discussed above, we queried whether AMPK or ERK might be candidate mediators of stilbenoid effects on the vasculature. We observed significantly increased phosphorylation of AMPKα at Thr172, which is an indicator of AMPK activation status [55,56], in SHHF mesenteric arteries (Figure 4A) but not cerebral vessels (Figure 4C). This increase in levels of phosphorylated AMPK is likely due in part to a trend where total AMPK levels are increased in untreated and resveratrol- or pterostilbene-treated SHHF rats (since statistical significant changes are obscured by normalization with total AMPK) (Figure 4B). In the presence of gnetol, AMPKα phosphorylation failed to reach statistical significance, whereas AMPKα phosphorylation remained elevated in SHHF mesenteric arteries despite the presence of resveratrol or pterostilbene. No differences in ERK activation were detected in mesenteric resistance nor middle cerebral arteries (Figure 5).

## 3. Discussion

To our knowledge, this is the first study to characterize the structural and mechanical properties of peripheral (i.e., mesenteric) and brain (middle cerebral) resistance arteries in the SHHF rat. The SHHF rat models human heart disease in that it superimposes risk of heart failure upon hypertension [47,48]. First, we detected bed-specific differences in the nature of arterial wall abnormalities in the SHHF rat. Although mesenteric and cerebral arteries exhibited identical differences in vascular geometry (increased media-to-lumen ratio) and compliance (reduced), disparate differences were detected in the development of these aberrations. For example, in mesenteric arteries from untreated SHHF, the increase in media-to-lumen ratio and virtually unchanged media CSA indicate eutrophic remodeling, which is supported by the calculated remodeling and growth indices of 97.4% and 3.9%, respectively. In contrast, SHHF middle cerebral arteries also exhibited an increase in media-to-lumen ratio, and a clear trend suggesting media CSA is on a trajectory of growth. This, too, is supported by calculated growth and remodeling indices of 43.6% and 58%, respectively. Therefore, in contrast to the sole eutrophic remodeling that occurred in mesenteric vessels, a combination of hypertrophic growth and eutrophic remodeling likely occurred in cerebral vessels. Another example of regional differences is wall component stiffness (slope of elastic modulus vs. stress), which was increased in SHHF cerebral arteries but not in mesenteric arteries. Vascular compliance, or the ability to buffer changes in pressure, is determined by a combination of geometry and wall component stiffness [57]. This suggests, therefore, that mere remodeling only produced a trend (*p* = 0.07) toward reduced compliance in SHHF mesenteric arteries, whereas in SHHF cerebral arteries, the presence of both remodeling and wall component stiffening led to a clear, statistically significant (*p* < 0.01) reduction in compliance.

This study is predicated, at least in part, by our previous reports that resveratrol attenuated remodeling and mechanical changes in mesenteric resistance arteries [33]. We hypothesized that we would likewise observe normalization of vascular abnormalities in the SHHF rat. In fact, stilbenoid treatment did attenuate increases in media-to-lumen ratio (mesenteric and cerebral arteries) and wall component stiffening (cerebral arteries). According to Table 1, attenuation of morphological changes likely involved attenuation of hypertrophic growth in middle cerebral arteries vs. eutrophic remodeling in mesenteric arteries. Notably, we also hypothesized that, because pterostilbene exhibits improved bioavailability and prolonged half-life compared to resveratrol [36,58], we would observe greater vascular effects with pterostilbene (and perhaps gnetol). However, despite the significant differences in oral bioavailability between resveratrol (20%), pterostilbene (80%) and gnetol (7%) [35,36,42], there were no differences between the efficacy of their effects on vasculature. This suggests that the stilbenoid compounds, and/or perhaps their bioactive metabolites [59], were indeed accessing both arterial beds to produce equivalent effects. Thus, contrary to our hypothesis, the purportedly improved oral bioavailability of pterostilbene [36,58] did not influence its effects on the vasculature compared to resveratrol or gnetol.

One outstanding question pertains to the contribution of stilbenoid-induced blood pressure lowering to vascular effects. Admittedly, we did not detect statistically significant reductions in blood pressure in response to resveratrol, pterostilbene, or gnetol. While this is consistent with our previous report on the lack of blood pressure effect (at least for resveratrol) in SHR [33], we would be remiss if we did not note a trend, at least for resveratrol, to reduce blood pressure. It is plausible that the high number of groups (and *n* values) in our study confounded the statistical power for SBP. Given first, the small (if any) blood pressure lowering effect, and second, the seemingly graded anti-hypertensive responses to resveratrol (~7 mm Hg), pterostilbene (~4 mm Hg) and gnetol (~2 mm Hg) versus the equivalent vascular responses, we speculate that improvement of vascular structure was a result of direct actions on the arterial wall rather than solely secondary to reduced hemodynamic stress derived from BP lowering. This would also be consistent with clinical reports showing that an increase in the media-to-lumen ratio predicts cardiovascular events independent of BP lowering [60,61]. Nonetheless, there may be value to investigating higher doses of resveratrol in a future study, particularly in adult rats with established hypertension. Indeed, doses ranging from 10–150 mg/kg/day attenuated hypertension in rats fed high-fat diets, [32] partially-nephrectomized rats [30], the fructose-fed rat [31], as well as angiotensin II-infused and SHR [62]. Thandapilly et al. reported that low dose resveratrol, when combined with hydralazine to reduce blood pressure, was more effective than either agent alone in terms of improving parameters of cardiac function and vascular remodeling [63]. We speculate that should a higher dose of resveratrol reduce blood pressure in the SHHF rat, we would see complete suppression of remodeling rather than moderate attenuation.

It also bears mentioning that the abnormalities observed in SHHF mesenteric resistance arteries differ from those previously reported for SHR. For example, mesenteric arteries from adult SHR exhibit a combination of hypertrophic and eutrophic remodeling [54,64], whereas we observed primarily eutrophic remodeling. Mechanical properties also differ between SHR and SHHF mesenteric arteries. Previous reports documented increased compliance [33] and reduced wall component stiffness [33,65] in SHR arteries, and these resemble the changes in subcutaneous small arteries from patients with mild essential hypertension [57]. In contrast, we report here a trend toward reduced compliance (or at least, not increased compliance as reported in SHR [33]) and unchanged wall component stiffness in mesenteric arteries from SHHF rats; whether these vascular aberrations model those found in the context of heart failure (risk) superimposed upon hypertension remains to be determined. Interestingly, the profile of cerebral changes is similar between SHR and SHHF rats, whereby cerebral arteries from both species stiffen [66].

This may be important because during hypertension, end-organ damage extends to various organs, such as the eyes [67] and kidneys [68], but the brain [69] is especially susceptible. Sustained high BP is an important risk factor for stroke [70,71], cognitive decline, and dementia [71,72]. The brain depends on the continuous supply of oxygen and energy substrates maintained by cerebral blood flow. In hypertension, regional cerebral blood flow is reduced over time and associated with functional decline in brain regions involved in memory [73]. Longitudinal studies generally indicate that increases in BP are associated with cognitive impairment [74]. Arterial stiffening is also related to cognitive decline [75], and is a better predictor of cognitive decline than BP [76]. Heart failure further potentiates the threat of cognitive decline [77] and promotes the progression of cognitive decline to *bona fide* dementia or Alzheimer’s disease [78]. Importantly, 75% of heart failure patients have pre-existing hypertension [79].

Blood is supplied to the brain by carotid and vertebral arteries which merge at the base of the brain to form the circle of Willis [80]. Arteries departing the circle of Willis carry blood along the brain surface, branching into pial arteries that branch further yet into arteries penetrating into the brain parenchyma. Arteries responsible for blood delivery to the brain are abnormal in models of cardiovascular disease, although in ways different from the peripheral microvasculature. In SHR and stroke-prone SHR (SHRSP), cerebral arteries (large supply arteries) and arterioles (small, resistance arteries) undergo hypertrophic remodeling [81,82,83] but become less stiff and more compliant due to changes in vessel wall composition (an increased ratio of (compliant) elastin to (stiff) collagen) [81,84]. In contrast, peripheral resistance arteries stiffen [54,85]; SHR mesenteric resistance arteries have a greater ratio of collagen to elastin [54]. This disparity suggests regional differences in vascular response. Such differences may also be diameter-dependent; small pial arterioles (30–75 µm) dilate at high (>170 mm Hg) intraluminal pressures to a greater extent than large pial arterioles (185–384 µm) which constrict [86].

Thus, our findings, that a combination of remodeling and wall component stiffening occurred in SHHF cerebral arterioles to reduce vascular compliance may provide insight to microvascular mechanisms that contribute to cognitive decline in patients with hypertension and risk of heart failure. It may be informative to interrogate other rat models, such as SHR and the SHR-SP rat, in the future.

In an effort to identify potential signaling mediators, we queried a potential role of AMPK. As found in SHR [49], SHHF also exhibit elevated activation status of AMPK within mesenteric arteries (although not in cerebral arteries); however, given the ability of stilbenoids to normalize media-to-lumen ratio in both arterial beds, and also the inability of resveratrol and pterostilbene to attenuate AMPK activation, it is unlikely that AMPK plays a major role. We also identified ERK as a potential effector of stilbenoid effects. First, mesenteric resistance arteries from adult SHR exhibit amplified ERK signaling, and growth responses in vascular smooth muscle cells from SHR were abolished by ERK inhibition [53]. Second, we reported that the ability of resveratrol to attenuate remodeling of SHR mesenteric arteries was associated with blockade of exaggerated ERK signaling [33]. Our data demonstrate, however, that ERK represents another difference between SHHF and SHR mesenteric arteries. Indeed, ERK activity, whether normalized by total ERK or β-actin, is not different between SHHF and SD arteries, and is unaffected by stilbenoids.

Failure to detect meaningful roles of AMPK and ERK led us to speculate which other signaling pathways might contribute to stilbenoid effects. We did not detect any meaningful changes in p38, JNK, nor oxidative status (Supplementary Materials Figures S1–S3). One possible player might be eNOS; however, Rush et al. reported that resveratrol improved nitric oxide (NO)–mediated vasorelaxation in SHR without altering eNOS expression [87]. Thus, rather than modulating eNOS levels (or activation status) *per se*, resveratrol might prevent free radical–induced degradation of NO, thereby rescuing or increasing NO-cGMP/PKG signaling. Consistent with this notion, we reported that, at least in SHR, resveratrol amplified phosphorylation of vasodilator-stimulated phosphoprotein (VASP) at serine 239, a reliable marker of PKG activity [33]. Future experiments might therefore interrogate stilbenoid effects on PKG activity in SD vs. SHHF arteries. Another candidate of interest is the TGFβ/SMAD pathway, given its contribution to vascular remodeling vis-à-vis upregulation of pro-fibrotic genes and therefore fibrosis [88,89]; however, upstream activation of ERK or p38 promotes TGFβ/SMAD [88,89], yet we failed to detect meaningful differences in ERK nor p38 that would support such a signaling axis. Finally, we are interested in activation of peroxisome proliferator-activated receptors (PPARs) as a potential target of stilbenoids. PPARs belong to the nuclear receptor family of transcription factors that regulate lipid metabolism [90]. Upon ligand activation, PPARs form heterodimers with the *retinoid X receptor* (*RXR*), and the PPAR-RXR heterodimer binds to *peroxisome proliferator response elements* (*PPREs*) in the promoter region of PPAR-regulated genes. PPARα and PPARγ mRNA are greater in young and adult SHR mesenteric arteries compared to WKY (but not in other tissues) [91], and thizaolidinedione PPARγ agonists attenuated remodeling and endothelial dysfunction in mesenteric resistance arteries in response to angiotensin II [92] or endothelin-1 [93]. Evidence suggests that phosphatidylinositol 3-kinase/Akt lies downstream of PPAR activation [94]. Direct binding occurs between resveratrol and PPARs α and γ [95,96], and it is generally accepted that resveratrol activates endogenous PPARs α and γ isoforms, but not PPARδ (even at 100 µM) [97], although resveratrol can activate PPARδ when overexpressed [98]. Thus, it would be interesting to interrogate PPAR levels in SHHF mesenteric and cerebral arteries, and to investigate whether a PPAR/PI3K/Akt signaling axis signals stilbenoid effects therein.

In conclusion, we report here for the first time that SHHF resistance arteries exhibit region-specific abnormalities (namely, remodeling [eutrophic] in mesenteric vessels vs. remodeling [eutrophic and hypertrophic] and stiffening in cerebral vessels). Stilbenoid treatment attenuated remodeling to similar degrees, despite first, a lack of statistically significant reductions in blood pressure, and second, the remarkedly greater bioavailability of pterostilbene compared to resveratrol and gnetol. These data suggest that these stilbenoids (and/or their metabolites) exerted, at least in part, direct actions on the vascular wall. Neither AMPK nor ERK serve as effectors of stilbenoid effects in the microvasculature; perhaps the anti-inflammatory and anti-oxidant actions instead effected the improvement [36,45,46]. It should be noted that the deleterious effects of hypertension extend to the brain. Indeed, hypertension is a leading risk factor for stroke [70,71], cognitive decline, and dementia [71,72], and anti-hypertensive pharmacotherapy reportedly attenuates cognitive decline [99,100]. Moreover, heart failure potentiates the threat of cognitive decline [77]. Notably, we report for the first time that in the SHHF model, where risk of heart failure is superimposed upon hypertension, middle cerebral arteries exhibit reduced compliance and wall component stiffening in addition to the vascular remodeling observed in a hypertensive model *per se.* We therefore speculate that cerebral microvascular disease might be an important contributor to cognitive decline, and may be an important therapeutic target to mitigate the risk of cognitive decline in the context of cardiovascular disease. This warrants further study.

## 4. Materials and Methods

### 4.1. Animals

This study was approved by the University of Manitoba Animal Care Committee (Protocol Reference Number: 14-056) and follows Canadian Council of Animal Care guidelines. Male SD and lean SHHF rats were obtained from Charles River (Senneville, QC, Canada) at 7 weeks of age. The use of SD rats as controls is predicated on the fact that SHHF animals were developed from cross breeding SHR-obese with SHR; the SHR-obese strain was bred using SD rats (normotensive) and SHR (hypertensive), while SHR were bred using WKY animals with above-average blood pressure [101,102]. Therefore, the SD rat is the appropriate normotensive control for the SHHF rat and as such, we have published studies using SD rats as normotensive control for SHHF rats previously [103,104]. Incidentally, it also bears mentioning that the use of WKY rats as controls for SHR is common practice, yet considered problematic due to issues such as genetic disparity within WKY [105] and biological variability [106]. Animals were housed under a 12-h light/dark cycle at 22 °C and 60% humidity and fed *ad libitum*.

Rats were trained for blood pressure measurement using tail cuff plethysmography (CODA non-invasive blood pressure system; Kent Scientific, Torrington, CT, USA), after 2 weeks of acclimatization and biweekly thereafter. SD and SHHF rats were treated for 8 weeks by oral gavage with vehicle (i.e., 50% ethanol) or equivalent doses (2.5 mg/kg/day; dissolved in 50% ethanol, as previously described [59,63,107]) of resveratrol, pterostilbene, and gnetol (Sigma Aldrich-Canada, Oakville, ON, Canada; Cayman Chemical, Ann Arbor, MI, USA; and kindly provided by Dr. Kalyanam Nagabhushanam (Sabinsa Corporation, East Windsor, NJ, USA), respectively. This dose was chosen based on our previous study that showed vascular improvement by resveratrol in SHR [33].

### 4.2. Pressure Myography

#### 4.2.1. Arterial Segments

At 17 weeks of age, systolic blood pressure (SBP) measurements were acquired. Rats were then anesthetized with isoflurane (initial: 5%, maintenance: 3%), received an injection of heparin (1000 U/mL; 1 mL/kg) via the saphenous vein, and were euthanized by terminal excision of the heart. The mesenteric cascade was isolated and placed in ice-cold Krebs buffer (mM: NaCl 118, KCl 4.65, MgSO_4_ 1.18, KHPO_3_ 1.18, NaHCO_3_ 25, CaCl_2_ 2.5, glucose 5.5, EDTA 0.26). To maintain consistency and ensure unbiased sampling, segments of mesenteric arteries were dissected from third-order branches. Middle cerebral arteries were dissected proximal to the internal carotid arteries. Arterial segments (mesenteric or middle cerebral) were mounted on two glass micro-cannulas and secured with nylon ties in a pressure myograph chamber (Living Systems Instrumentation, Burlington, VT, USA) such that the walls were parallel without stretch. Vessels were then equilibrated for 1 h at constant intraluminal pressure (45 mm Hg and 30 mm Hg, respectively) at 37 °C with aerated Krebs buffer (20% O_2_ and 5% CO_2_) to obtain pH 7.4. Vessels were considered viable if >50% constriction was elicited with KCl (125 mM).

#### 4.2.2. Vascular Geometry

Vessels were deactivated by bath-perfusing the artery with Ca^2+^-free Krebs solution containing 1 mM EGTA for 30–60 min. Lumen and media dimensions were measured at three points along the length of the vessels at constant intraluminal pressure (mesenteric arteries, 45 mm Hg; middle cerebral arteries, 30 mm Hg).

#### 4.2.3. Vascular Mechanics

Intraluminal pressure was raised from 3 to 140 mm Hg three times, and arteries were unbuckled by adjusting the cannulas. Initial diameters were measured at 3 mm Hg. Pressure-lumen diameter relationships were obtained by incrementally increasing the intraluminal pressure from 3 to 140 mm Hg. Lumen and media measurements were measured in triplicate along the length of the vessel at each pressure increment, and subsequently used to calculate mechanical properties of vascular walls, as described below.

#### 4.2.4. Formulas

Media cross-sectional-area (CSA) was calculated by the subtraction of the internal CSA from the external CSA: π(D_e_^2^ − D_i_^2^)/4, where D_e_ and D_i_ are external and internal diameters, respectively. Media strain, which reflects pressure-induced changed in diameter, was calculated as ε = (D − D_o_)/D_o_, where D is the internal diameter for a given intraluminal pressure, and D_o_ is the baseline diameter at 3 mm Hg. Media stress was determined by σ = P × D/2 × M, where P is the intraluminal pressure, D is the internal diameter and *M* is the media thickness. Pressure is converted as 1 mm Hg = 1.334 × 10^3^ dyn/cm^2^. The elastic modulus (EM) was determined by fitting the stress-strain data to the exponential equation (y = ae^bx^) using least squares analysis: σ = σ_o_e^βε^, where σ_o_ is stress at the baseline diameter, D_o_, and β is a constant related to the rate of increase in the stress-strain curve. Tangential elastic modulus (ET) was calculated at several values of stress from the derivative of the abovementioned exponential curve: ET = dσ/dε = βσ_o_e^βε^. The slope of the EM versus stress curve reflects the intrinsic stiffness of the wall components. Remodeling index is the percentage difference in the lumen diameters of hypertensive and normotensive vessels that is due to remodeling, 100 × [(D_i_)_n_ − (D_i_)_remodel_]/[(D_i_)_n_ − (D_i_)_h_], where (D_i_)_n_ and (D_i_)_h_ are mean lumen diameters of normotensive and hypertensive vessels, respectively, and (D_i_)_remodel_ is [(D_e_)_h_^2^ − 4 × CSA_n_/π)]^0.5^, where (D_e_)_h_ is the external diameter of hypertensive vessels. Growth index is the percentage of the difference in the lumen diameters of hypertensive and normotensive vessels that is due to hypertrophy, (CSA_h_ − CSA_n_)/CSA_n_, where CSA_n_ and CSA_h_ are mean media CSAs of normotensive and hypertensive vessels, respectively.

### 4.3. Western Blotting

Arterial lysates were prepared in RIPA buffer, clarified by centrifugation, and p-AMPK (Cell Signaling Technology (Whitby, ON, Canada)), native AMPK, p-ERK and native ERK (Cell Signaling Technology, Danvers, MA, USA) were detected by conventional western blotting. As applicable, membranes were stripped and reprobed with β-actin antibody to account for loading variations among lanes.

### 4.4. Statistics

Data are expressed as mean ± SEM. Statistical analysis of data was performed by, as applicable, applying one-way analysis of variance (ANOVA) or two-way ANOVA for repeated measures, followed by Bonferroni *post-hoc* tests for multiple comparisons. *p* < 0.05 was considered significant.

## Figures and Tables

**Table 1:** Blood pressure and stiffness of mesenteric and middle cerebral arteries in 17 week old SD and SHHF rats: effect of stilbenoids.

Parameter	SD				SHHF			
	C	R	P	G	C	R	P	G
body weight, g	564 ± 15	574 ± 19	552 ± 24	600 ± 38	375 ± 10 **	363 ± 14 **	351 ± 12 **	364 ± 9 **
systolic BP	142 ± 6	132 ± 7	136 ± 3	142 ± 5	194 ± 3 **	187 ± 5 **	190 ± 3 **	192 ± 4 **
mesenteric arteries—slope of EM vs. stress	4.4 ± 0.2	4.9 ± 0.3	4.2 ± 0.1	4.5 ± 0.1	5.0 ± 0.3	5.3 ± 0.4	5.3 ± 0.3	5.4 ± 0.4
cerebral arteries—slope of EM vs. stress	6.1 ± 0.9	6.0 ± 0.7	5.9 ± 0.6	5.7 ± 1.3	18.2 ± 2.9 **	11.8 ± 1.1 *^,†^	9.4 ± 1.1 ^††^	11.5 ± 1.0 ^†^

C—control, R—resveratrol, P—pterostilbene, G—gnetol. * *p* < 0.05, ** *p* < 0.01 vs. SD controls, and ^†^ *p* < 0.05; ^††^ *p* < 0.01 vs. untreated SHHF.

**Table 2:** Remodeling and growth indices of mesenteric and middle cerebral arteries in 17 week old SHHF rats vs. SD rats: effect of stilbenoids.

Arteries	Growth Index				Remodeling Index			
	C	R	P	G	C	R	P	G
Mesenteric arteries	3.9%	5.6%	19.4%	14.0%	97.4%	96.2%	79.9%	90.2%
Middle cerebral arteries	43.6%	4.3%	7.0%	-10.3%	58.0%	54.5%	48.6%	39.0%

C—control; R—resveratrol; P—pterostilbene; G—gnetol. Media CSA was normalized for weight (SHHF BW/SD BW)^0.05^ [54].

## Supplementary Materials

Supplementary materials are available online.
