# Supplementary material for: Peripheral and Cerebral Resistance Arteries in the Spontaneously Hypertensive Heart Failure Rat: Effects of Stilbenoid Polyphenols
Source: Molecules. 2017 Feb 28;22(3):380. doi: 10.3390/molecules22030380 (PMC6155253; doi:10.3390/molecules22030380)
Supplement: Supplementary file 1 [file molecules-22-00380-s001.pdf]

Supplementary Materials: Peripheral and Cerebral Resistance Arteries in the Spontaneously Hypertensive Heart Failure Rat: Effects of Stilbenoid Polyphenols

Danielle I. Lee, Crystal Acosta, Christopher M. Anderson, Hope D. Anderson

p38 and JNK are not likely effectors of stilbenoid-dependent microvascular effects.

Arterial lysates were prepared in RIPA buffer and clarified by centrifugation. p-p38 and p38 (Cell Signaling Technology, Danvers, MA), as well as p-JNK and JNK (Santa Cruz Biotechnology, Dallas, TX, USA) were detected by conventional western blotting. As applicable, membranes were stripped and probed with actin antibody to account for loading variation among lanes.

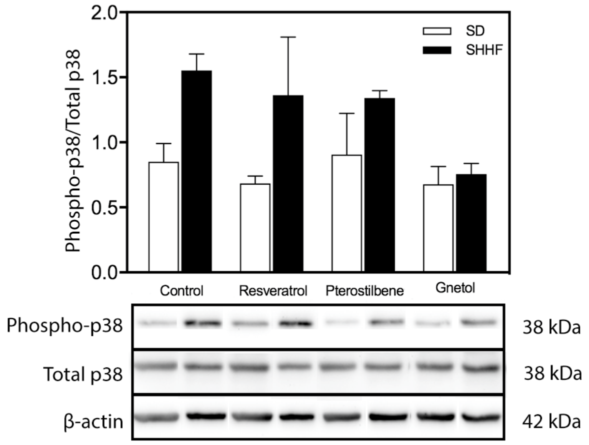

Figure S1. Phosphorylation status of p38 is similar between SD and SHHF mesenteric arteries, in the presence and absence of resveratrol, pterostilbene, or gnetol. $n = 3$.

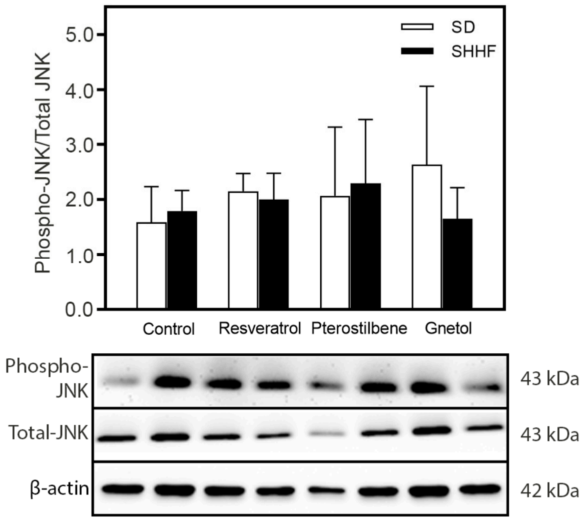

Figure S2. Phosphorylation status of JNK is similar between SD and SHHF cerebral arteries, in the presence and absence of resveratrol, pterostilbene, or gnetol. $n = 3$.

Abnormal oxidative stress is not evident in plasma from SHHF vs. SD rats.

To determine lipid peroxidation in plasma, an assay kit to measure malondialdehyde (MDA; Abcam Toronto, ON, Canada) was used, as per the manufacturer's protocol. Absorbance of samples was measured at 532 nm using a FLUOstar Omega Microplate reader (BMG Labtech, Guelph, ON, Canada).

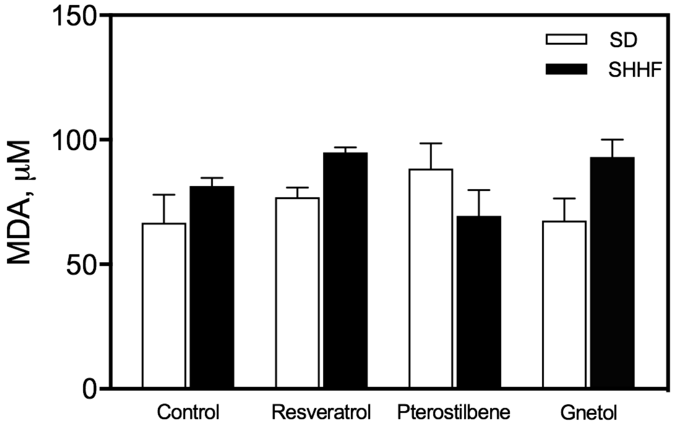

Figure S3. Plasma oxidative status is similar between SD and SHHF rats, in the presence and absence of resveratrol, pterostilbene, or gnetol. $n = 3$.
